# Pituitary Tumour Apoplexy as Cause of Death of Simonetta Vespucci, the *Venus* by Botticelli

**DOI:** 10.1002/edm2.70261

**Published:** 2026-06-12

**Authors:** Domiziana Nardelli, Dennis Black, Anne Schafer, Paolo Pozzilli

**Affiliations:** ^1^ Department of Endocrinology and Diabetes Università Campus Bio‐Medico Rome Italy; ^2^ Department of Epidemiology and Biostatistics University of California, San Francisco San Francisco California USA; ^3^ Blizard Institute Queen Mary University of London London UK

**Keywords:** galactorrhea, pituitary adenoma, tumour

## Abstract

**Introduction:**

Simonetta Vespucci is the model for the world‐renowned Venus painted by Sandro Botticelli at the end of the XV Century. Her unexpected death at the age of 23 remained a mystery for more than 500 years, until we proposed in 2019 that she probably suffered from a pituitary adenoma.

**Methods:**

We now hypothesize with further evidence from historical descriptions of her last days that an expansion of the adenoma was the cause of her premature death, aged twenty‐three.

**Results:**

Our hypothesis is supported by three elements: (1) tumour characteristics; (2) symptoms presented during her last days; and (3) the historical records of two potential precipitating events.

**Conclusions:**

We conclude that Simonetta Vespucci, the Venus by Botticelli, died as a consequence of a rapidly expanding pituitary adenoma causing tumour apoplexy, thereby making this endocrine emergency the likeliest cause of her death.

## Graphical abstract

A reassessment of Simonetta Vespucci, muse of Sandro Botticelli, attributes her long‐mysterious death to a pituitary adenoma. Clinical reinterpretation of historical accounts suggests tumour expansion led to sellar destruction, severe haemorrhage, and pulmonary complications, offering a coherent medical explanation for her sudden death at twenty‐three.

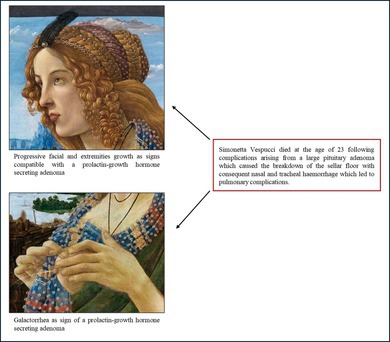

Simonetta Vespucci, born Cattaneo (Liguria, January 28th 1453), was an undisputed protagonist of Italian Renaissance: she is the *Venus* portrayed in the *Birth of Venus* (1482–1485) by Sandro Botticelli.

In 1469, she married Marco Vespucci, descendant of a noble family of Florentine bankers. With this union Simonetta joined Florentine society, under the Signoria de'Medici. Given her fine manners and intellect, she bewitched the greatest Florentine minds, as the humanist Poliziano, who gave her the title of La Sans Par (‘the Unrivalled’). Among them all, she became intimately close to illustrious patrons of the arts Lorenzo and Giuliano de'Medici.

Sandro Botticelli was perhaps her most devoted admirer and close friend. Simonetta's features embodied the aesthetic canons of the Renaissance, inasmuch as the painter immortalized her beauty even long after her tragic death. In 1476, shortly before her premature demise, Simonetta collapsed and fell prey to a copious epistaxis while attending a ball. Professor Strano reconstructed the occurrence: ‘she laid helpless, for a little or for a long time, in absence of light, unable to react, while the bystanders tried to stop the blood running from her nose’ [1]. Within days, her health rapidly deteriorated, leaving her bedridden.

Through an exchange of letters between Piero Vespucci and Lorenzo de'Medici, we can characterize Simonetta's last days. She suffered from copious epistaxis, rhinorrhea, debilitating headaches and confusion; she even appeared to hallucinate at times, together with vomiting spells and high fevers [2]. Lorenzo de'Medici promptly sent his physician, Maestro Stephano, to tend his beloved Simonetta. He believed her illness was intrinsic, influenced by living in Vespucci's mansion. Prominent physician to the Vespucci family, Maestro Moyse also attended Simonetta and found himself disagreeing with Stephano, attributing symptoms to the *likeliest* cause: consumption. After a long dispute, they agreed to administer some medicine against consumption, which unfortunately had no effect.

Simonetta died in April, 1476, aged twenty‐three.

Her body was exposed to public veneration, *vestita e scoperta* (dress in white, face uncovered); the highest homage to illustrious Renaissance figures. She still rests in the church of Ognissanti, wherein 1510 Sandro Botticelli would ask to be buried at her feet, last devotional act to his Muse.

## 1 Delving Deeper: An Expanding Adenoma

**FIGURE 1:**
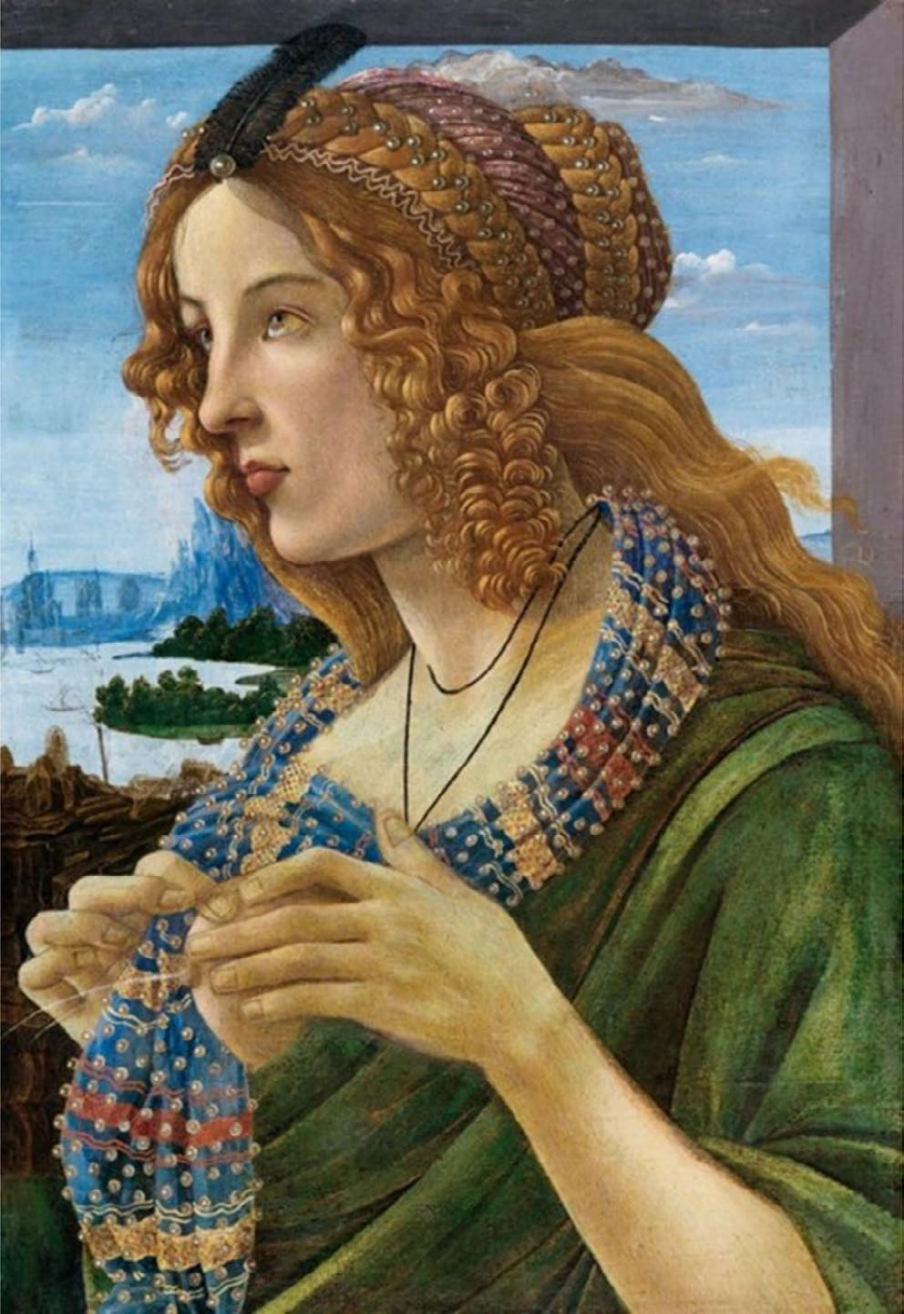
Allegorical portrait of a woman (Simonetta Vespucci), Sandro Botticelli (1480–1490 ca). Canvas, Private collection.

The *Allegorical portrait of a Woman* (1476 ca) by Botticelli portrays a woman (Figure 1), later identified as Simonetta, with a stream of milk running from her right breast, facing away as if repulsed: a surprising depiction for a likely infertile woman. Historically, she never gave birth. Moreover, Marco Vespucci remarried after her death and had nine children, indicating he was a fertile man [3].

Galactorrhea and a history of infertility, together with apparent changes in facial traits, led Pozzilli et al. speculate that Simonetta suffered from a prolactin‐growth hormone secreting adenoma [3].

This diagnosis was confirmed through a facial recognition algorithm based on a pre‐trained deep learning model, verifying that the progressive facial growth portrayed by Botticelli across five portraits of Simonetta is compatible with facial changes observed in patients with secreting adenomas [3].

Given her adenoma diagnosis, Simonetta's symptoms may reflect a tumour expansion through the sphenoid sinus and nasal cavities.

Silva Junior et al. described a patient with a history of galactorrhea, amenorrhea; progressive facial and extremities growth, which are signs compatible with a prolactin‐growth hormone secreting adenoma [4]. Specifically, the hypothesis of a macroadenoma eroding the sphenoid sinus and invading the nasal cavities was supported by episodes of epistaxis and rhinorrhea, with this acute presentation being the fil‐rouge drawing his case together with Simonetta.

It's important to note that the patient, like Simonetta, had no visual impairments, an element typically associated with the *local mass effect* of growing macroadenomas.

This made our hypothesis of an adenoma expanding exclusively through the sphenoid and nasopharynx a plausible scenario, even in the absence of historical records mentioning any visual disturbance.

Yet, observation of the *Birth of Venus* (1482–1485) leaves space to some speculation on a potential visual disturbance. Here, Simonetta is depicted with a characteristic irregular eye positioning, commonly known as ‘strabismus of Venus’. Large macroadenomas may cause diplopia and ophthalmoparesis by cranial nerve compression in cavernous sinus [5]. However, it is unclear whether strabismus was a feature of Simonetta herself, rather than a trait of piety and beauty attributed to the goddess Venus. Actually, during the following century, exaggerated features like strabismus will be much appreciated hallmarks of Mannerism [6, 7].

Relying on attained symptoms alone, ectopic pituitary adenoma (EPA) must be included into differential diagnosis, being clinically indistinguishable from pituitary adenomas due to their hormone‐secreting nature. Peng et al. have reported a case study of epistaxis and pituitary apoplexy due to a ruptured internal carotid artery aneurysm embedded within pituitary adenoma [8]. Agely et al. [9] illustrated a case of a forty‐eight‐year‐old woman complaining of confusion, headaches, fever and rhinorrhea, stemming from a sphenoidal ectopic prolactinoma. Therefore, it is impossible to further narrow our differential diagnosis in the absence of imaging tools.

## 2 Simonetta's Last Days

Her adenoma grew undisturbed over the course of several years, until such a voluminous mass may have generated irreparable damage to the delicate vascular network supplying the pituitary gland, causing hemorrhagic or ischemic infarction. An important differential diagnosis when identifying intratumoral haemorrhage is pituitary apoplexy, which corresponds to a rare yet potentially life‐threatening clinical syndrome characterized by the sudden onset of symptoms such as severe headache, vomiting, visual disturbances, ophthalmoplegia, altered mental status, and possible panhypopituitarism. This condition predominantly occurs in individuals with hemorrhagic infarction of the pituitary gland, often associated with an existing pituitary macroadenoma. While certain pathological and physiological conditions may present with similar imaging features, a combination of clinical presentation and imaging characteristics can help radiologists arrive at an accurate diagnosis, particularly through the use of MRI [10]. These phenomena are collectively known as pituitary tumour apoplexy (PTA) [11]. All of the above‐mentioned signs and symptoms are compatible with a picture of PTA, thereby making this endocrine emergency the likeliest cause of death.

Since Simonetta died at 23, data from a younger population may best reflect her presentation. In a retrospective study carried out by Jankowski et al. [12] of nine young patients affected by PTA, the most common symptoms were headache (100%), dizziness (44%) and visual impairment (66%), including blurred vision and photophobia [12], which likely afflicted Simonetta, since she preferred to rest in a dark room during her last days.

There is no univocal mechanism subtending PTA etiopathology. However, there are two most credited theories [13], both of which can be applied to Simonetta's case. The first theory is that the growth pace of the adenoma outstripped that of angiogenesis, while the second one correlates with the pituitary stalk effect, leading to progressive constriction of vascular supply. Larger tumours have a greater tendency to bleed. Moreover, prolactinomas are the most prone to PTA among the secreting adenomas [13, 14, 15, 16, 17]. On top of that, head trauma is a validated precipitating factor for PTA [18].

Two main factors may have precipitated her clinical picture: firstly, she frequently attended balls as a Vespucci family representative, since dance was considered the finest display of education during the European Renaissance. The genre of the *alte danze* [19], entailed quick movements and jumps. Considering that she collapsed during a ball, the mechanical trauma of repeated jumps endured by anatomical structures already compromised by an expanding mass may have hastened the onset of haemorrhage. The second factor concerns encounters between Simonetta Vespucci and Alfonso II D'Aragona, Duke of Calabria. He grew close to the Signoria in 1473, when his sister, Eleonora D'Aragona, joined the duke of Ferrara, Ercole I d'Este, in a second marriage. There, he met Simonetta through the de'Medici family.

He was infamously known for his lack of morals. The anonymous author of the *Chronicum Venetum* portrays him as a perpetrator of ‘most cruel insults and abuses’, rape and ‘taking the women of others for his own pleasure’, though the historical accuracy is rather doubtful.

Theologist Lorenzo Sardi, in his poem *De Anima Peregrina*, hints that Simonetta was ambushed by the man on the banks of the river Arno as she was seeking relief from the summer heat [19].

Considering that the above‐mentioned encounter credibly occurred after 1473, the hypothesis of a mechanical trauma following the violent encounter remains a plausible precipitating factor. Even an abrupt change in blood pressure caused by great distress could have induced apoplexy of such a voluminous tumour alone [20].

## 3 Conclusions

With a known prolactinoma‐growth hormone secreting adenoma, evidence is sufficient to support progression in the natural history of the tumour, leading to an expansion that preferably involved the sphenoid sinus and nasal cavities, whereby sparing the visual pathways.

Readers must be aware that reports of such dramatic pituitary adenomas are rare in current medical literature, thanks to the use of laboratory tests and imaging tools, allowing diagnosis and treatment in the early stages. Ectopic pituitary adenomas must be included in the differential diagnosis, since it is not possible to infer the anatomical origin in the absence of imaging tools.

In conclusions, pituitary tumour apoplexy was Simonetta's likeliest cause of death, based on three elements:

1. tumour characteristics: dimensions, expansion and prolactin secretion;
2. symptoms experienced during her last days;
3. the hypothesis of two precipitating events: repeated mechanical trauma caused by dancing and a possible violent encounter.

More than 500 years after their dispute, Maestro Stephano's intuition was not wrong, demonstrating that unconventional possibilities are worth exploring, in addition to the epidemiologically‐expectable diagnoses of the time.

## Author Contributions

**Domiziana Nardelli:** conceptualization, writing – original draft, writing – review and editing, validation. **Paolo Pozzilli:** conceptualization, writing – review and editing, writing – original draft, validation. **Dennis Black:** conceptualization, writing – original draft, writing – review and editing, validation. **Anne Schafer:** conceptualization, writing – review and editing, writing – original draft, validation.

## Funding

The authors have nothing to report.

## Disclosure

The authors have nothing to report.

## Ethics Statement

The authors have nothing to report.

## Conflicts of Interest

The authors declare no conflicts of interest.

## Data Availability

The data that support the findings of this study are available from the corresponding author upon reasonable request.
